# Impaired mRNA Expression of the Migration Related Chemokine Receptor CXCR4 in Mesenchymal Stem Cells of COPD Patients

**DOI:** 10.1155/2017/6089425

**Published:** 2017-07-19

**Authors:** K. Karagiannis, A. Proklou, E. Tsitoura, I. Lasithiotaki, C. Kalpadaki, D. Moraitaki, I. Sperelakis, G. Kontakis, K. M. Antoniou, N. Tzanakis

**Affiliations:** ^1^School of Medicine, Laboratory of Molecular and Cellular Pneumonology, University of Crete, Heraklion, Crete, Greece; ^2^Department of Respiratory Medicine, University Hospital of Heraklion, Heraklion, Crete, Greece; ^3^Department of Haematology, University Hospital of Heraklion, Heraklion, Crete, Greece; ^4^Department of Orthopedics, University Hospital of Heraklion, Heraklion, Crete, Greece

## Abstract

Defective tissue repair and remodeling are main aspects of Chronic Obstructive Pulmonary Disease (COPD) pathophysiology. Bone marrow mesenchymal stem cells (BM-MSCs) have been implicated in this direction, as their functional impairment and recruitment could possibly contribute to disease development and progression. The present study characterizes for the first time the expression of migration related chemokine receptors and their ligands in BM-MSCs from COPD patients. CXCR4/SDF1a and CCR7/CCL19-CCL21 mRNA levels were evaluated in BM-MSCs obtained from twelve COPD patients and seven healthy donors. SDF1a protein levels in sera and BM-MSCs' conditioned media were also evaluated. CXCR4, SDF1a, CCL19, and CCL21 mRNA levels were significantly reduced in COPD BM-MSCs while CCR7 levels were undetectable. Notably, SDF1a protein levels were marginally elevated in both patient sera and BM-MSCs' conditioned media while the increase in SDF1a serum levels significantly correlated with disease severity in COPD. Our findings show posttranscriptional regulation of SDF1a levels in BM-MSCs of COPD patients and significant downregulation of SDF1a and CXCR4 mRNA indicating an involvement of the SDF1a signaling pathway in the disease pathophysiology.

## 1. Background

Chronic Obstructive Pulmonary Disease (COPD) is defined as a “common preventable and treatable disease characterized by persistent airflow limitation that is usually progressive and associated with an enhanced chronic inflammatory response in the airways and the lung to noxious particles or gases.” Exacerbations and comorbidities contribute to the overall severity in individual patients [1]. COPD is one of the leading causes of death globally, with huge social and economic burden [2]. Thus, it is estimated to be the fifth most common cause of morbidity and the third leading cause of death worldwide by 2030 [3]. Tobacco smoking, being well established as the main etiological factor for the development of COPD, induces a vicious circle of epithelial damage, impaired tissue repair, and remodeling. However, despite the plethora of research studies in COPD, the exact pathophysiological mechanisms are not yet fully understood.

Mesenchymal stem (stromal) cells (MSCs) are multipotent stromal cells that can be isolated from bone marrow, skeletal muscle, amniotic fluid, and adipose tissue, among others tissues. They are plastic adherent cells with a differentiation potential towards adipocytes, chondrocytes, and osteoblasts [4]. Waterman et al. have recently proposed that MSCs may be polarized into a proinflammatory MSC1 population or immunosuppressive MSC2 phenotype, dependent on their exposure to specific stimuli [5]. Therefore, it is likely that MSCs play a crucial role in both the maintenance of epithelial integrity and the pathogenesis of lung diseases. During the last few years a number of experimental data on animal models have showed MSCs' contribution to tissue regeneration in certain lung diseases such as elastase-induced emphysema, homing to sites of asbestos-induced lung injury, tissue remodeling in pulmonary hypertension, decrease in chronic airway inflammation in asthma, and restoration of alveolar and lung fluid balance after endotoxin-induced acute lung injury [6–11]. Consequently, great interest in the therapeutic potential of ex vivo expanded MSC has been noticed for the treatment of many disease states [12–14]. Bone marrow–derived MSCs are activated and migrate to sites of injury via mechanisms that have not yet been fully elucidated. The proposed mechanism of attraction at the sites of lung injury is via the production of soluble factors such as granulocyte colony-stimulating factor and granulocyte-macrophage colony-stimulating factor, which lead to MSC proliferation and migration via the stromal cell derived factor- (SDF-) 1–CXCR4 axis [15, 16]. Similarly, previous data from Antoniou et al. showed overexpression of CXCR4 in patients with Idiopathic Pulmonary Fibrosis (IPF) [17]. Additionally, CCR7 and its ligands CCL19 and CCL21 have recently been suggested as another chemotactic axis for MSCs [18, 19]. Hence, changes in chemokines and their receptors expression by stem cells can represent defects on the migratory ability of those cells.

The aim of the current study is to investigate the possible involvement of BM-MSCs in the pathophysiology of COPD by evaluating the expression of genes involved in proliferation/migration process, namely, the CXCL12a/CXCR4 and CCR7/CCL19, CCL21 ligand/receptor dyad.

## 2. Methods

### 2.1. Ethics and Patient Data

This study was conducted in accordance with the amended Declaration of Helsinki. The Research Ethics Committee of the University of Crete approved the study protocol and written informed consent was obtained from the 19 subjects before entering the study (Decision 412, 18/06/2014 protocol number 14724). Of these, the 12 participants diagnosed as having COPD, according to the GOLD guidelines, were current or ex-smokers who had at least a 20-pack-year smoking history and had an FEV1/FVC ratio of <70% and reversibility to an inhaled beta-2 agonist of <10% or <200 mls absolute improvement [20]. Six healthy smoking individuals, defined as smokers without airflow limitation, and one healthy ex-smoker, with an FEV1 predicted above 80%, were recruited and matched for age and for smoking history, as closely as possible.

### 2.2. Lung Function Tests

Lung function testing, including spirometry with a bronchodilation test and DLCO measurement, was performed by all participants with a computerized system (MasterLab; 2.12, Jaeger, Wuerzburg, Germany) according to standardized guidelines.

### 2.3. Blood Samples Collection

Peripheral blood samples and serum were obtained from all participants and were stored at −80°C, after appropriate preparation, according to standard procedures.

### 2.4. BM-MSCs Collection and Culture

BM-MSCs were obtained from posterior iliac crest aspirates and cultured in vitro as previously described [17, 21]. In brief, BM mononuclear cells (BMMCs) isolated with Histopaque-1077 (Sigma, Saint Louis MO) were cultured in Dulbecco's Modified Eagle Medium-Low Glucose (DMEM-LG; Gibco Invitrogen, Paisley Scotland)/10% fetal calf serum (FCS; Hyclone, Logan, Utah, USA)/100 IU/ml penicillin-streptomycin (PS, Gibco) (MSC medium) at a concentration of 2 × 10^5^ cells/cm^2^ in 25 cm^2^ culture flasks in 37°C temperature/5% CO2 humidified atmosphere. One to three days after seeding, floating cells were removed and the medium was replaced by fresh MSC medium. Cells were passaged when 70–90% confluence was reached, using 0.25% trypsin-1 mM EDTA (Gibco). MSCs were identified by their morphologic and immunophenotypic characteristics and their potential to differentiate towards three different pathways, namely, adipocytes, osteocytes, and chondrocytes.

### 2.5. Immunophenotypic Characteristics of MSCs

BM-MSCs from second passage (P2) were immunophenotypically characterized by flow cytometry using anti-human monoclonal antibodies against anti-CD73 (AD2; Becton Dickinson-Pharmingen, San Diego, CA), anti-CDw90 (F15.42; Immunotech/Coulter), anti-CD105 (SN6; Caltag, Burlingame, CA), anti-CD45 (IMMU19.2; Immunotech/Coulter), anti-CD14 (IM2580U; Beckman-Coulter), and antiCD34 (QBend10; Beckman-Coulter). Data were processed in an Epics Elite flow cytometer (Coulter, Miami, FL).

### 2.6. Real-Time Reverse Transcriptase-Polymerase Chain Reaction Assay

BM-MSCs at P2 were homogenized in TRIzol reagent (Invitrogen, Carlsbad, CA), total RNA was extracted, and cDNA was synthesized by reverse transcription (RT) with the Thermoscript RT kit (Invitrogen). mRNA expression was measured using a real-time RT-PCR assay with SYBR-Green I. Glyceraldehyde-3-phosphate dehydrogenase (GAPDH) was used as the internal control, in order to normalize SDF-1a, CXCR4, CCR7, CCL19, and CCL21 expression levels. Relative expression levels per sample were calculated as 2^−(Ct  of  gene  of  interest − Ct  of  GAPDH)^. The mRNA-specific primers used are listed in Table 1.

### 2.7. Chemokine Protein Levels in Serum and BM-MSCs Conditioned Media

SDF-1a levels in MSC conditioned media at P2 and sera were evaluated by means of ELISA (Quantikine; R&D Systems, Minn., MN).

### 2.8. Statistical Analysis

Results were analyzed using IBM SPSS statistics v21.0. Nonparametric Mann–Whitney statistical test was used and values were expressed as medians (interquartile range). Spearman's rank coefficient (rho) was used to evaluate correlations between severity of the disease (FEV1% pred) and chemokines expression. A value of *p* < 0.05 was considered as statistically significant.

## 3. Results

### 3.1. Demographics and Lung Function

Table 2 displays patients' data. There were no significant differences between COPD and control group in age, gender, smoking status, and history and Body Mass Index (BMI). Lung function testing results are shown in Table 3. As expected, there were statistically significant differences between COPD patients and controls in forced expiratory volume in one second (FEV1%, 49% versus 107%, resp., *p* < 0.001), forced vital capacity (FVC%, 64% versus 103%, resp., *p* = 0.001), and FEV1/FVC ratio (60% versus 79%, *p* < 0.001).

### 3.2. BM-MSCs Identification and Immunophenotypic Characteristics

BM-MSCs from both COPD and healthy subjects were cultured to P2 and subsequently were characterized by their immunophenotypic characteristics and differentiation capacity. Immunophenotypic analysis demonstrated the presence of a homogenous cell population positive for CD73, CD90, and CD105 and negative for CD45, CD14, and CD34 antigens (Table 4). Additionally, MSCs were found capable of differentiating towards the adipogenic, osteogenic, and chondrogenic lineages.

### 3.3. SDF1a/CXCR4 Axis

**Figure 1:**
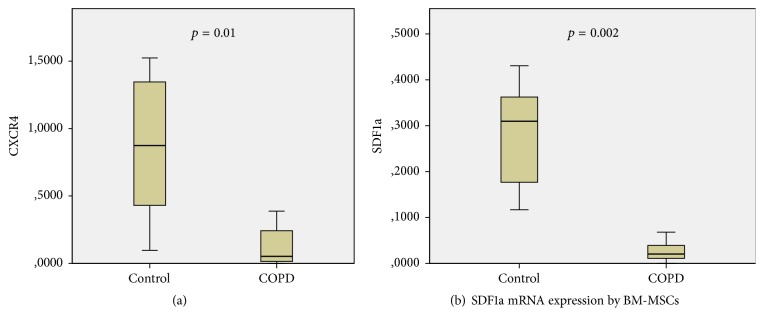


CXCR4 mRNA levels were reduced in BM-MSCs of COPD patients, as Figure 1(a) displays. Particularly, a significant downregulation of CXCR4 expression was detected in COPD in comparison with control group (0.05 versus 0.87 relative expression levels, resp., *p* = 0.01, Table 5).

BM-MSCs SDF1a mRNA levels were also significantly reduced in COPD relative to healthy subjects (0.03 versus 0.32 relative expression levels, resp., *p* = 0.005, Figure 1(b), Table 5). Interestingly, SDF1a protein levels in the BM-MSCs conditioned media were found increased in the COPD group, albeit nonstatistically significant (5570 versus 2995 pg/ml, *p* = 0.053).

**Figure 2:**
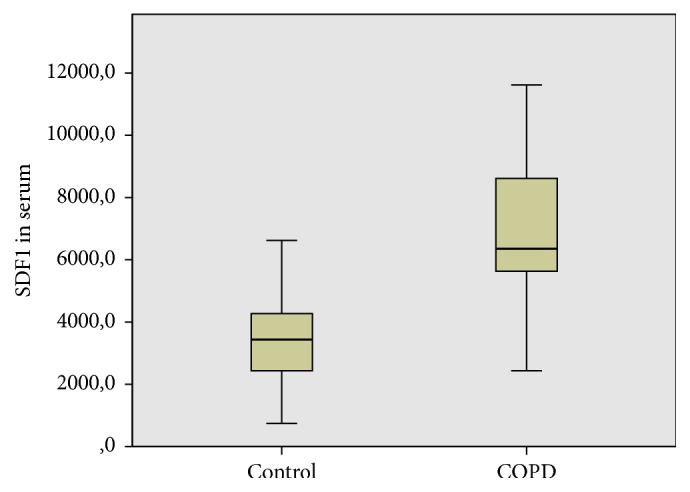
SDF1a protein levels in serum.

Additionally, serum SDF1a levels were higher in COPD patients as compared to healthy subjects; however, our results did not reach statistical significance (6358 versus 3941 pg/ml, *p* = 0.079, Figure 2).

**Figure 3:**
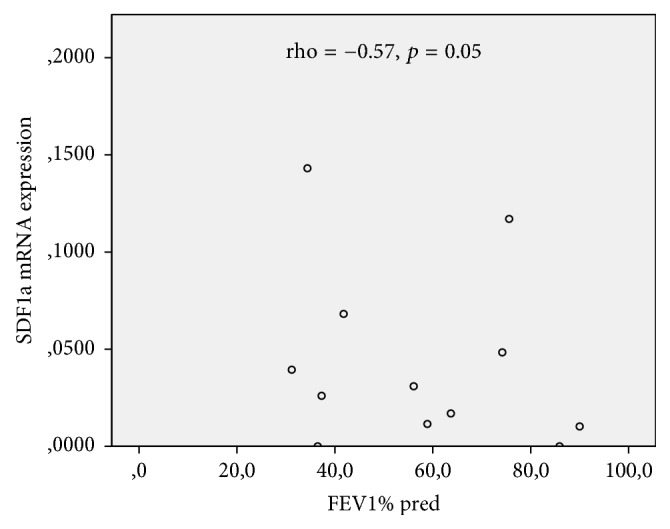
Correlations between SDF1a levels and severity of disease.

Notably, both SDF1a mRNA expression by BM-MSCs and serum protein levels were positively correlated to the severity of disease (GOLD stages) (rho = 0.59, *p* = 0.04 and rho = 0.63, *p* = 0.03, resp.). Expectedly, SDF1a mRNA levels were inversely correlated to FEV1% predicted of COPD patients (rho = −0.57, *p* = 0.05, Figure 3).

### 3.4. CCR7/CCL19-CCL21 Axis

**Figure 4:**
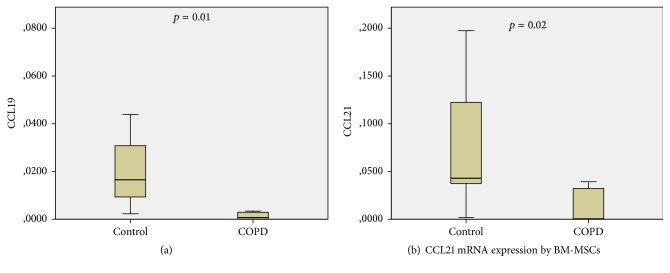


Real-time RT-PCR showed no detectable CCR7 mRNA expression in both groups. Nevertheless, its ligands' expression was downregulated in BM-MSCs of COPD patients. Specifically, CCL19 (0.001 versus 0.02, *p* = 0.01, Figure 4(a)) and CCL21 (0.00 versus 0.04, *p* = 0.02, Figure 4(b)) mRNA levels were significantly reduced in COPD BM-MSCs compared to controls (Table 5).

## 4. Discussion

Our study aims to characterize the expression of chemokines and chemokine receptors involved in the migration of mesenchymal stem cells such as the SDF1/CXCR4 and CCR7/CCL19 CCL21 axes, in BM-MSCs of COPD patients in comparison to healthy subjects with similar age and smoking histories. Interestingly, our results showed decreased CXCR4 mRNA expression by BM-MSCs obtained from COPD patients suggesting a possible impairment of their migratory capacity. Similarly, mesenchymal cells from the COPD group expressed lower mRNA levels of SDF1a, CCL19, and CCL21 compared to controls; however, within the patient group SDF1a mRNA positively correlated with disease severity. Additionally, SDF1a serum protein levels were found elevated in the patient group and positively correlated to the severity of disease (GOLD stages) suggesting activation of SDF1a pathway in COPD. Our study suggests therefore a role of CXCR4/SDF1a axis in COPD pathophysiology that may lead to deregulation of the migration ability of stem cells from bone marrow.

Chronic inflammation and defects in repair mechanisms are considered key mechanisms in COPD development and progression of [22, 23]. Recently, mesenchymal stem cells have been used in several animal and human studies as a potential therapeutic option for the disease, due to their anti-inflammatory and repair effect [24–27]. Our team has previously investigated the role of SDF1/CXCR4 axis in migration of BM-MSCs in Idiopathic Pulmonary Fibrosis and Rheumatoid Arthritis with Usual Interstitial Pneumonia [17, 28]. Adachi et al. showed that bone marrow transplantation had curative effect in mice with emphysema while transplantation from emphysematous mice induced the disease in normal ones, indicating a major role of bone marrow in the pathophysiology of this disease [29].

Stem cell exhaustion and senescence are considered potential pathogenetic mechanisms in COPD [30]. It could be hypothesized that an impairment of the migratory capacity of BM-MSCs could have a proinflammatory effect in COPD patients. Our results show a reduced expression of CXCR4 mRNA in BM-MSCs of COPD patients compared to healthy subjects. However, we did not find an inverse correlation between CXCR4 mRNA expression and COPD stage. Several researchers have demonstrated the importance of CXCR4 expression by mesenchymal stem cells for the mediation of their trafficking [31–33]. Recently, Yang et al. have demonstrated that CXCR4 overexpression by MSCs improves homing and colonization of damaged lung tissue and additionally suppresses the acute lung injury in animal models [31]. In another study, Bustos et al. showed that aged BM-MSCs have reduced migratory and anti-inflammatory capacity and CXCR4 is one of the genes that are downregulated in these cells [32]. The reduced mRNA expression of CXCR4 in BM-MSCs of COPD patients in our study may therefore provide evidence of a defect in migratory properties of these cells and therefore a reduced ability to mobilize and play their protective role. Liu and Xie have demonstrated that endothelial progenitor cells from COPD patients decrease in numbers and show impaired migration ability, specifically lower levels of CXCR4 expression [34]. To the best of our knowledge, the present study is the first study providing evidence that COPD affects CXCR4 mRNA expression of BM-MSCs.

Our results also showed an increase in SDF1a serum levels of COPD patients, although statistical significance was not reached. Activation of the SDF1a pathway could be expected due to inflammation and tissue damage in the lung of COPD patients and our finding of elevated SDF1a levels could point to this direction; however this hypothesis requires further investigation. This assumption, further supported by the positive correlation of SDF1a serum levels with COPD stages, indicates an association of protein levels to the severity of the disease. SDF1 secretion from sites of tissue injury results in the increased migratory rates of progenitor cells and BM-MSCs [18, 35, 36]. Zhang et al. have demonstrated that SDF1 and Hypoxia Inducible Factor-1a (HIF-1a) are involved in regulation of MSCs function and homing to the lungs of COPD rat models [37]. Our study demonstrates that SDF1a is also induced in COPD in humans.

Findings for SDF1a expression by cultured BM-MSCs are intriguing. SDF1a mRNA expression by COPD mesenchymal cells was significantly reduced compared to controls but protein levels in conditioned media were higher in COPD group. Our data imply the presence of a posttranscriptional regulation of SDF1a production by BM-MSCs in COPD. It is known that MSCs are expressing SDF1 but its role has not yet been fully elucidated [38]. Lisignoli et al. have demonstrated an inverse correlation between CXCR4 and SDF1 expression by MSCs, suggesting a suppressive autocrine loop for CXCR4 expression [39]. A similar finding was reported by Pelekanos et al. in their study of fetal MSCs, but autocrine loop for CXCR4 expression was not adequate to fully explain their results [40]. It seems that there are multiple molecular pathways implicated in SDF1a expression and secretion by MSCs. Interestingly, SDF1a mRNA expression within the COPD group showed a positive correlation with severity of the disease similarly to the serum levels of SDF1a. This result is confirmed, as expected, by a significant inverse correlation of SDF1a mRNA levels with FEV1% predicted. Both mRNA expression and secretion of SDF1a by MSCs are affected in COPD patients, according to the aforementioned data; it appears though that further characterization of the regulation of SDF1a expression is required.

Another chemotactic axis that has been studied as a possible regulator of mesenchymal stem cell migration is CCR7 and its ligands CCL19 and CCL21 [41]. Our results showed downregulation of both CCL19 and CCL21 mRNA transcripts in COPD patients similarly to SDF1a, whereas there was no detectable expression of CCR7 in both COPD and control BM-MSCs. Although not extensively investigated, CCR7/CCL19/CCL21 axis has been also implicated in MSCs chemotaxis and tissue repair [18]. Demoor et al. have demonstrated that CCR7 plays a major role in modulating inflammatory responses in airways in COPD, and cigarette smoking upregulated CCR7, CCL19, and CCL21 mRNA expression in lymph nodes of wild-type mice [42]. CCL19 and CCL21 mRNA levels were found significantly lower in COPD BM-MSCs, similarly to the CXCR4 and SDF1a mRNAs. This reduction in mRNA levels may also be associated with the senescence of BM-MSCs previously suggested as a pathogenic mechanism in COPD [30].

There are limitations in our study, which need mentioning. First, the limited sample size due to the practical difficulties of patient recruitment is noteworthy. However, this was a pilot/guide study, investigating ex vivo BM-MSCs from COPD patients which, based on our results, should be expanded to a larger patient group. Additionally, since COPD is a heterogeneous disease, different disease stages may be reflected on BM-MSCs properties and should be taken into consideration in future studies. Considering that cigarette smoking may affect the migration of BM-MSCs even in subjects without COPD, another limitation of our study is the lack of a group of healthy nonsmokers. A future study comparing the migratory capacity of BM-MSCs between smokers and nonsmokers could provide new knowledge about the effect of cigarette smoking on stem cells.

In conclusion, our study demonstrates a downregulation of the CXCR4 mRNA expression by BM-MSCs in COPD patients, providing for the first time evidence from a human study that CXCR4/SDF1 axis is dysregulated in COPD. Additionally, it shows that COPD also affects SDF1a levels in serum and BM-MSCs. Although further studies are needed, we suggest that potential defects in migratory capacity of BM-MSCs may compromise their protective role, leading to development and progression of COPD.

## Figures and Tables

**Table 1:** Primer sequences used for real-time RT-PCR.

Gene		Primer sequence (5′-3′)	Annealing temperature
CXCR4	FOR	GGTGGTCTATGTTGGCGTCT	55°C
	REV	TGGAGTGTGACAGCTTGGAG	
SDF1a	FOR	TGAGAGCTCGCTTTGAGTGA	55°C
	REV	CACCAGGACCTTCTGTGGAT	
CCR7	FOR	GGTGGTGGCTCTCCTTGTCATTTT	55°C
	REV	AGTTCCGCACGTCCTTCTT	
CCL19	FOR	GGACTTCCCCAGCCCCAACTCT	62°C
	REV	TAACTGCTGCGGCGCTTCATCTT	
CCL21	FOR	CCTCAGCTCTGGCCTCTTAC	57°C
	REV	GGGGCAAGAACAGGATAGCTG	
GAPDH	FOR	AGCCACATCGCTCAGACA	53°C
	REV	CCAATACGACCAAATCCGTT	

**Table 2:** Patient's demographic data.

	COPD patients (*N* = 12)	Controls (*N* = 7)	*p* value
Age (years)	65.5 (55.3–72)	58 (33–63)	NS
Gender (m/f)	11/1	6/1	NS
BMI	30.1 (259–31.8)	27.8 (25.5–31.1)	NS
Smoking status (c/ex)	7/5	6/1	NS
PY	53 (40–78.8)	30 (12–80)	NS
COPD Gold stage			
Stage 1	2		
Stage 2	4		
Stage 3	5		
Stage 4	1		

Values are expressed as median (interquartile range) or frequencies; *p* value < 0.05 as statistically significant; m/f = male/female; BMI = Body Mass Index; c/ex = current/ex-smokers; PY = pack years.

**Table 3:** Lung function test results.

	COPD patients (*N* = 12)	Controls (*N* = 7)	*p* value
FEV1%	49 (35–72)	107 (80–120)	<0.001
FVC%	64 (56–81)	103 (87–120)	0.001
FEV1/FVC ratio	60 (45–70)	79 (75–83)	<0.001
RV%	165 (141–204)	105 (79–139)	0.045
TLC%	112 (99–123)	98 (94–112)	NS
RV/TLC ratio	56 (50–69)	37 (28–45)	0.001
DLCO%	65 (27–94)	82 (72–96)	NS
KCO%	84 (43–110)	90 (77–106)	NS

Values are expressed as median (interquartile range); *p* value < 0.05 as statistically significant; FEV1 = forced expiratory volume in 1 second; FVC = forced vital capacity; TLCOc = transfer factor of the lung for carbon monoxide; KCO = transfer coefficient; RV = residual volume; TLC = total lung capacity.

**Table 4:** Flow cytometry results.

	CD 73	CD 90	CD 105	CD 45	CD 14	CD 34
MSCs	98.2 (1.6)	97.8 (1.65)	97.8 (1.9)	0.4 (1.77)	0.3 (1.45)	0 (0)

Values are expressed as median (interquartile range).

**Table 5:** Real-time RT-PCR results.

	COPD patients (*N* = 12)	Controls (*N* = 7)	*p* value
CXCR4	0.05 (0.01–0.31)	0.87 (0.34–1.35)	0.01
SDF1a	0.03 (0.01–0.06)	0.32 (0.18–0.43)	0.002
CCL19	0.001 (0.00–0.03)	0.02 (0.004–0.04)	0.01
CCL21	0.00 (0.00–0.04)	0.04 (0.04–0.19)	0.02
CCR7	No expression	No expression	NS

Values are expressed as median (interquartile range); *p* value < 0.05 as statistically significant; measurements are 2^−DCT^; DCT= Ctgene – Ctgapdh.
